# Use of Rapid Qualitative Analysis to Support Collaborative Synergy Within a Community Coalition for Health Equity in Detroit

**DOI:** 10.1089/heq.2024.0160

**Published:** 2025-01-29

**Authors:** Hayley S. Thompson, Ten-Niah Kinney, Carrie Leach, Alexandra Sass, Ariel Washington, Rhonda Dailey, Elizabeth Towner, Alyssa Beavers, Rodlescia Sneed, Karen Solomon Edwards, Ijeoma Nnodim Opara, Arthur Hampton, Zachary Cichon, Afsana Rinky, Joneigh Khaldun

**Affiliations:** ^1^ Department of Oncology, Karmanos Cancer Institute, Wayne State University School of Medicine, Wayne State University, Detroit, Michigan, USA.; ^2^ Department of Oncology, Wayne State University School of Medicine, Detroit, Michigan, USA.; ^3^ Institute of Gerontology, Center for Urban Responses to Environmental Stressors (CURES), Wayne State University, Detroit, Michigan, USA.; ^4^ Department of Family Medicine and Public Health Sciences, Wayne State University School of Medicine, Office of Community Engaged Research (OCEnR), Wayne State University, Detroit, Michigan, USA.; ^5^ Department of Family Medicine and Public Health Sciences, Wayne State University School of Medicine, Detroit, Michigan, USA.; ^6^ Department of Nutrition and Food Science, Wayne State University College of Liberal Arts and Sciences, Detroit, Michigan, USA.; ^7^ Department of Psychology, Wayne State University College of Liberal Arts and Sciences, Institute of Gerontology, Wayne State University, Detroit, Michigan, USA.; ^8^ Detroit Community Health Equity Alliance (D-CHEA) Wayne State University Center for Health Equity and Community Engagement (CHECK-UP) , Detroit, Michigan, USA.; ^9^ Internal Medicine-Pediatrics, Wayne State University School of Medicine, Detroit, Michigan, USA.; ^10^ Southeastern Michigan Health Association, Detroit, Michigan, USA.; ^11^ CVS Health®, Woonsocket, Rhode Island, USA.

**Keywords:** health equity, community coalition, qualitative research, urban health, rapid qualitative analysis

## Abstract

**Introduction:**

A community coalition is an effective strategy for addressing complex health challenges. A citywide coalition of community and academic experts was formed to address Detroit’s persistent health disparities. To foster collaborative synergy, we explored hyperlocal perspectives on health equity by applying rapid qualitative analysis (RQA) as a time-efficient and rigorous approach.

**Methods:**

Twenty coalition members completed a key informant interview addressing five key areas: health equity meanings, Detroit’s most pressing health problems, social ecological domains that influence health equity and outcomes, and strategies to achieve health equity. We used RQA to interpret interview data.

**Results:**

Participants were majority female, Black/African American, and over 60 years old. Participants defined health equity as equal access to opportunities for a healthy life and emphasized the importance of individual choice in pursuing those opportunities. As an indication of their awareness of social determinants of health, participants articulated connections between various social ecological factors and health outcomes.

**Discussion:**

This study highlights participants’ recognition of both systemic factors and personal agency in achieving health equity, indicating their nuanced understanding of the complex interplay between social structures and individual health, which is crucial for community-driven multilevel health interventions. Furthermore, by fostering better communication and alignment, RQA is an efficient and effective method to enhance coalition synergy.

**Health Equity Implications:**

By facilitating a shared understanding of health equity and its determinants, RQA can help coalitions ensure inclusion and integration of diverse perspectives in intervention planning and delivery, particularly in urban settings facing similar challenges.

## Introduction

The creation of a community coalition, a group of individuals representing diverse organizations, factions, or constituencies within a community who agree to work together to achieve a common goal,^1^ is a compelling and widely used approach to addressing health challenges within a specific geographic area. Detroit, Michigan is a prime candidate for this approach. Despite its resilience, the city confronts longstanding population health disparities, including chronic disease mortality rates that are considerably higher than state and national averages (MDHHS, 2022).^2^ Systemic racism plays a significant role in the population health disparities observed there. According to the 2022 U.S. Census, almost 80% of Detroit residents identify as Black.^3^ Furthermore, Detroit is the most segregated U.S city by several metrics^4^ as a result of redlining practices, historical racial housing discrimination, and “white flight” from the city following the racial unrest associated with the 1967 Detroit riot/rebellion. These factors are linked to decades of disinvestment, leaving 33.8% of Detroiters burdened by poverty compared to 13.4% of Michigan residents.^5^ Recent public health practice in Detroit is complicated by the city’s financial struggles and efforts to privatize and outsource core public health department functions,^6^ a shift that coincided with the city’s 2013 bankruptcy filing.^7^ Regaining control of public health services in 2014 allowed Detroit to begin rebuilding its public health infrastructure. Despite this progress over the past decade, continued investment is needed to more fully address Detroit’s health disparities.

The Detroit Community Health Equity Alliance (D-CHEA) represents one effort to address local population health disparities. Evolving from a Black health and racial equity research workgroup, D-CHEA is a citywide coalition formed in 2023 comprised of community and academic experts committed to implementing multilevel community-based health interventions to improve cardiovascular health, cancer control, and mental health. D-CHEA is a multisector coalition representing community development organizations, public health associations, mental health service agencies, population-specific community-based organizations, including those serving and advocating for parents, older adults, and LGBTQ individuals, policy groups addressing food access/security and environmental justice, and private corporations.

In-depth understanding of the creation, sustainability, and effectiveness of community coalitions is facilitated by Community Coalition Action Theory (CCAT), which outlines constructs and practice-proven propositions related to the formation, maintenance, and institutionalization of coalitions.^8^ One such construct is collaborative synergy: the mechanism through which coalitions gain a collaborative advantage by engaging diverse members and pooling member, community, and external resources.^8,9^ Collaborative synergy depends on resources that are often described in material terms, such as donations, dues, or in-kind contributions.^8^ However, we posit that resources can also be intellectual, with sharing of ideas, conceptual frameworks, and intervention models. Lasker et al.^10,11^ have described this as strengthened thinking, a crucial component in coalitions since both individuals and organizations tend to possess limited or incomplete information. Coalitions foster ongoing dialogue among individuals with diverse types of knowledge, allowing the collective to overcome knowledge gaps and leading to more comprehensive thinking.^10,11^ Strengthened thinking may also involve more creative thinking, as dialogue within coalitions includes exploration of differences and challenging of conventional wisdom, leading to opportunities to break new ground.^10,11^

After D-CHEA was established and the coalition began to meet regularly, members expressed a need for intellectual collaborative synergy, asserting that it was imperative that the group build consensus around a working definition of health equity and develop a shared understanding of local social determinants. The work presented here describes our efforts toward these goals. Specific aims were to (1) explore hyperlocal perspectives on the definition of health equity, (2) characterize Detroit’s most urgent health problems and structural inequities that contribute to health inequity through a community lens, and (3) identify effective approaches to tackling these problems from the perspective of local stakeholders. The current report also describes the process and outcomes of qualitative key informant interviews conducted to uncover these perspectives and inform the coalition’s next steps. We applied rapid qualitative analysis (RQA) to this work, a pragmatic approach that facilitates the collection and analysis of targeted data in a shorter timeline than traditional qualitative methods while maintaining rigor.^12^ A final aim was to describe the ways in which data collection efforts and findings shaped the coalition’s plans and work in the short term.

## Methods

### Participants and Procedures

**FIG. 1.**
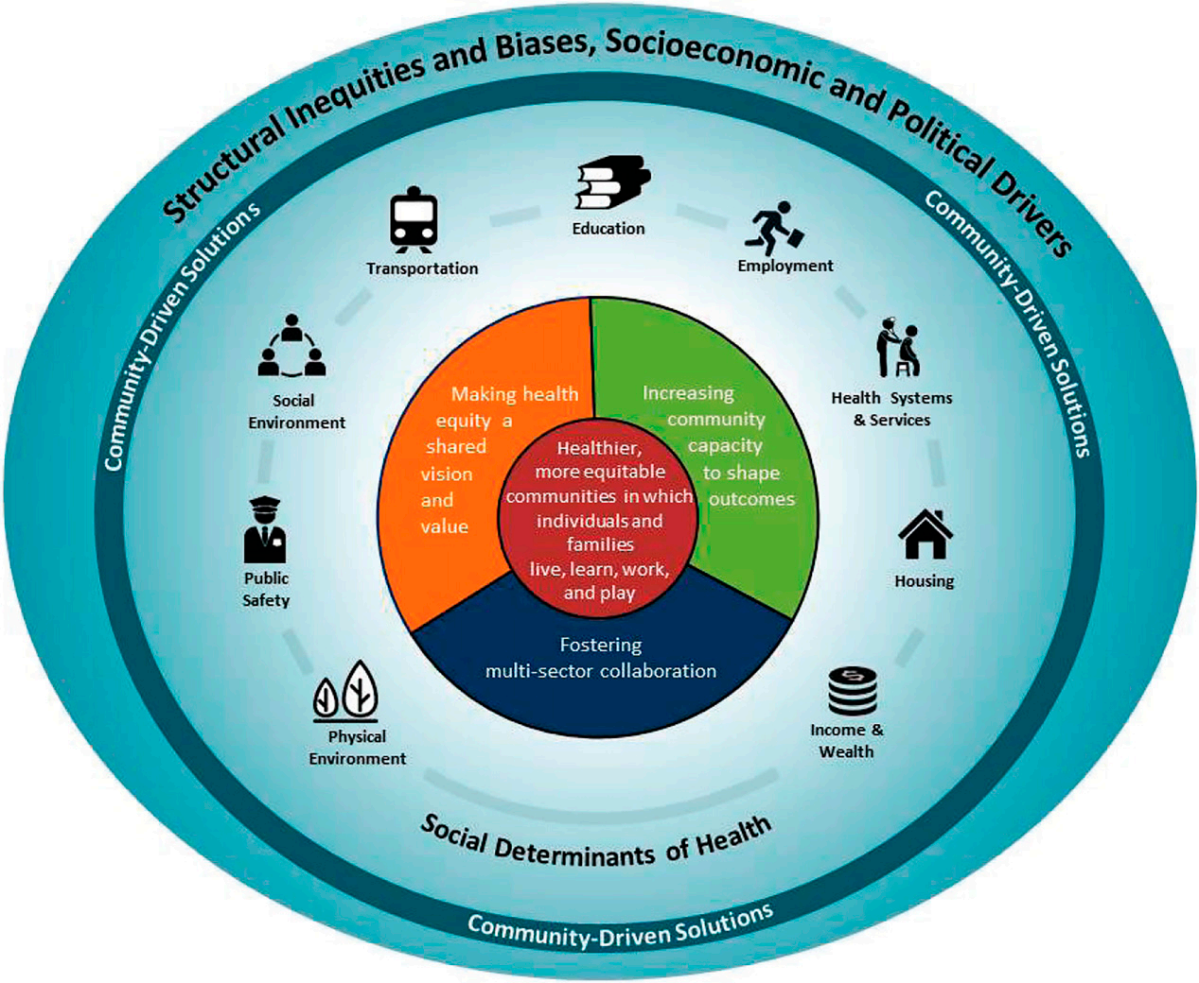
The Community-Based Solutions to Promote Health Equity Model.

Participants were recruited from the 23 non-academic coalition members who represented community interests. Informed consent was obtained from all the participants involved in this study. Participants were emailed a copy of the interview questions and a link to a brief demographic questionnaire prior to the interview. Interviews were 90 minutes in length on average and conducted by four research study team members via Zoom videoconferencing software between October 2023 and March 2024. All interviews were recorded and transcribed within the Zoom videoconferencing platform. Transcripts were then reviewed for accuracy and deidentified. The interview guide was informed by two frameworks visually shown to participants during the interviews. The first was the Community-Based Solutions to Promote Health Equity Model^13^ (Fig. 1), describing nine social determinants of health (SDOH) and expanded by the research team to include a tenth: the digital environment, as digital inclusion (e.g., internet connectivity, broadband access, digital literacy) has been characterized in previous work as a super determinant of health due to the ways in which it influences a multitude of other life domains. These include one’s ability to find and leverage resources related to economic sustainability, navigate one’s neighborhood and physical environment, identify educational opportunities, access food, build and maintain community and social networks, and manage health care.^14,15^

**FIG. 2.**
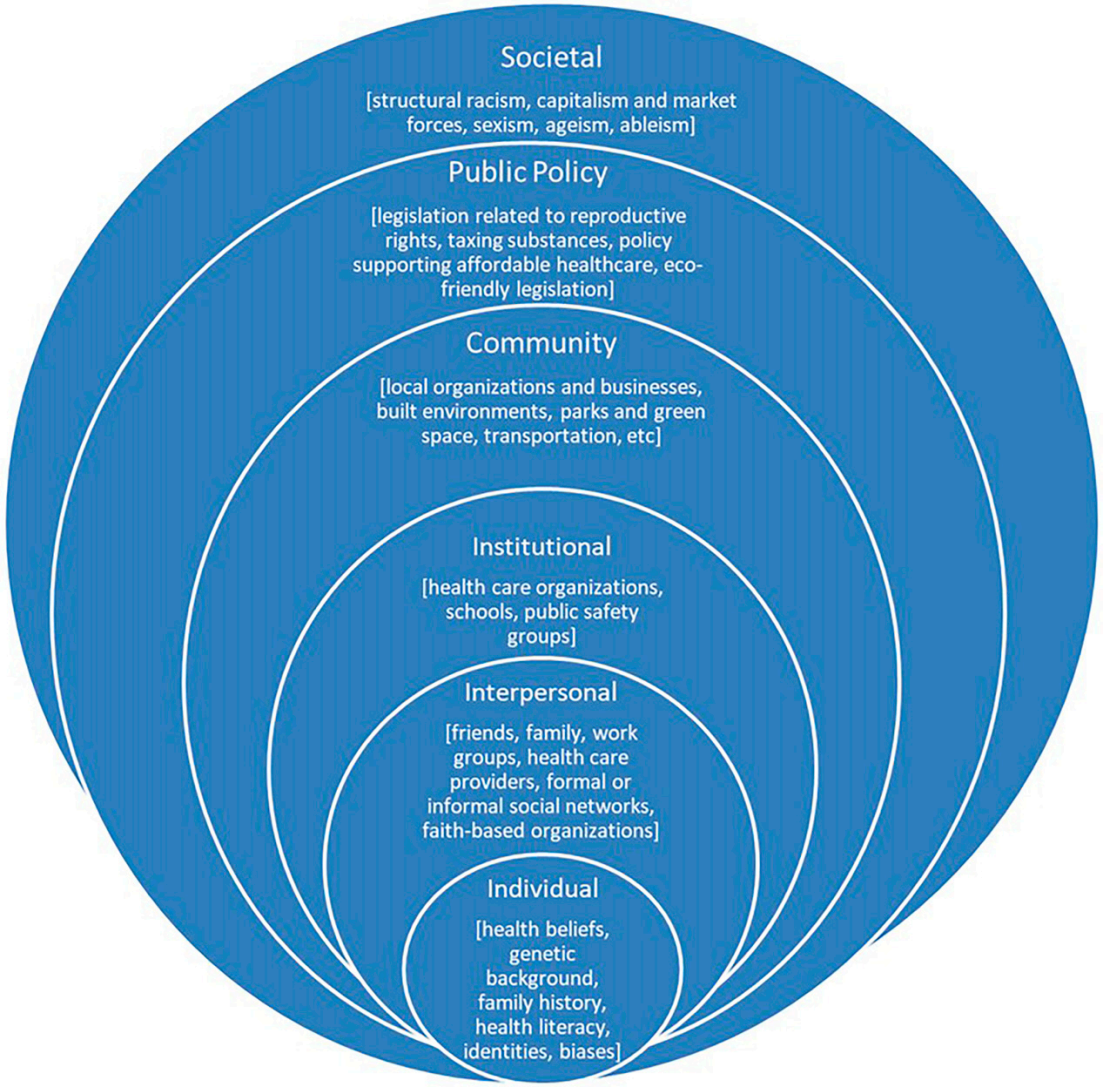
The Social Ecological-Model.

The second framework was the Social-Ecological Model (SEM), conceptualizing multiple levels of influence on individual, community, and population health (Fig. 2).^16^ This report focuses on five questions in the interview guide specific to the meaning of health equity, individual perceptions of the most pressing health problems facing Detroit, the most urgent SDOH that need to be addressed in Detroit, their beliefs about what areas are the most influential on health equity and health outcomes in Detroit based on the Social-Ecological Model, and awareness of strategies to achieve health equity. This study was approved by the Institutional Review Board at Wayne State University.

### Data Analysis

To analyze transcripts, we used Rapid Qualitative Analysis (RQA), an approach that facilitates analysis of targeted data in a shorter timeline than traditional qualitative methods while maintaining rigor.^12^ Lewinski et al.^12^ note that this approach is appropriate when data collection targets, such as selection of interviewees and expected deliverables, and processes, such as interview protocols, are highly structured. A high degree of structure was present in the current study given both the narrow range of participants (coalition members) and topics for exploration. Therefore, RQA as a focused, action-oriented approach was deemed appropriate for this study. RQA is often described as a purposeful data reduction strategy and “a form of analysis that sharpens, sorts, focuses, discards, and organizes data in such a way that ‘final’ conclusions can be drawn and verified.”^17^ In RQA, key points from qualitative data are summarized within matrices, making data more manageable and enabling more efficient exploration of relevant themes.^18^

The current analyses were based on established RQA practices.^19,20^ The team first created neutral domain names that aligned with each interview question. A standard summary template was then developed to capture each domain, with areas to add corresponding summaries. Next, transcripts were divided across four interviewers who extracted, verbatim, those parts of a participant’s response most relevant to the domain. In this step, Hamilton suggests that it is important to “keep the words” and avoid stripping the data from its original context.^19^ Team members then developed summaries of the extracted data. Based on recommendations for RQA, all team members “tested” and evaluated the summary template using the same subset of transcripts to ensure that domains were intuitive and readily located in the data.^19^ This step also allowed the team to compare the summarization style of each member and ensure that summaries were similar in length and depth. Once this step was complete, the team then analyzed the remaining transcripts, with each transcript reviewed by one team member. To establish rigor and validity, each transcript and its summary template were reviewed by a second coder who provided further elaboration or expansion as warranted. All summaries were then transferred to a matrix: a spreadsheet containing numerous cells into which summarized data are entered, domain (row) by participant (column).^21^ This resulted in a data display that allowed team members to easily compare and contrast responses across participants as well as within participant cases.^21^

## Results

### Participant Characteristics

In total, 20 (87%) of the 23 potential participants completed interviews: 15 organizational representatives (75%) and 5 neighborhood residents (25%) largely identifying as female (75%) and Black/African American (75%), either alone or along with other racial groups and slightly over half (55%) were >60 years of age. Each participant was assigned a unique study ID number that is noted after each direct quote presented as part of results.

### Definitions of Health Equity

Participant responses emphasized equal access to environments that support health and wellbeing, the opportunity to experience a high quality of life with one’s needs met, and the elimination of systemic barriers to wellbeing, specifically those associated with SDOH. Equal access to health care settings and providers was included, with the caveat that they are unbiased and responsive to community needs. Participants emphasized the importance of personal choice and agency in achieving wellbeing, including the ability to self-advocate, take care of oneself and one’s family, and contribute to the broader community. Participants maintained that health equity includes access to diverse pathways to wellbeing ***and*** the right to decide which pathway to pursue:

“Health equity to me means everyone has an opportunity for the healthiest journey possible that they choose. It’s exposure, it’s availability. But I don’t force you to make the decision. I expose you.” [P1005]

This was consistent with other responses indicating that health equity includes an individual’s power to create change. Finally, participants emphasized that health equity must be connected to a belief in every person’s intrinsic value:

“Health equity is when people believe in themselves and believe that they’re valuable, and when they believe that they’re valuable they will stand up, speak up for themselves, and I’m going to use the term, **demand** that they receive the respect and they receive the attention that they need.” [P1002]

### Urgent Health Problems in Detroit

Three types of responses emerged when participants were asked about the most urgent or pressing health problems in the city. The first category of response described specific medical conditions including diabetes and other metabolic disorders, cardiovascular disease and hypertension, asthma, cancer, lung disease, sexually transmitted infections, infant mortality, and COVID-19. Risk factors like exposure to lead and other environmental toxicants were discussed as well. Mental and behavioral health were noted as a concern, as were several population-specific problems: Black maternal health, cardiovascular disease among Black men, LGBTQ+ populations and trauma, older adult mobility, and youth suicidality.

The second category of response focused on SDOH rather than specific medical conditions. Leading responses referenced poverty and unemployment.

“…growing up here… We were a solid middle-class, working-class community. It was solid, but over the course of my lifetime, as educational attainment has declined and even educational opportunity, we have seen our poverty rate in our - my - neighborhood grow. So, we’ve become poorer and poorer… the opportunities to be able to find work where you can support yourself and your family…those opportunities have decreased as well, for a lot of reasons. …in a place like an inner-city neighborhood in Detroit, we see persistent poverty. Really, really, that’s just part of the system.” [P1001]

In addition, participants discussed challenges imposed by the social environment including food insecurity (e.g., food access, high food costs, and poor nutrition), public safety, in general, and the lack of safe space for physical activity. Additionally, participants described SDOH such as unaffordable or inaccessible medical and mental health care, transportation barriers, pollution, environmental racism, digital inequity, and the digital divide. Aligned with these concerns was the prioritization of daily survival over preventive health care and behaviors.

“It’s that we are in emergency, urgent versus preventative. And for the African American community, it’s cultural. When you’re in poverty, health isn’t your first priority - it’s surviving. And if I woke up, I have health. I don’t care what kind of health I have. I have health. I’m fighting for getting to the next day. And I’m not always thinking about my health unless it’s such a pain that it’s debilitating. [P1005]

On the individual level, adverse childhood events and limited exposure to modeling of healthy behaviors was mentioned, as was health literacy. Importantly, several participants emphasized the pathways through which SDOH leads to psychological and physiological stress which, in turn, activates disease pathways:

“…our body responds in ways to our environment stressors, you know. We’re built certain ways. But then that triggers these chemical processes that can deter health, or you can get sick, or you can develop disease, whether that’s cardiovascular health or diabetes, or, you know, just from the stress alone…” [P1001]

The third category of response focused on describing health care experiences, such as poor patient–provider communication (including reluctance to share one’s own social and economic challenges with one’s health care provider), medical mistrust, non-adherence to provider recommendations, and overburdened health care systems.

“I’ve been to every health system in the city of Detroit and I am not pleased. During my [illness], I spent more time in the hospital than I did at home for two years and I hated to go because when you went to the hospital and the waiting room you waited, waited, and waited, waited forever. And then when they get you into the emergency room, then you have to wait and wait and wait and wait for a bed. That’s not health equity. Our systems are broken in the United States and the COVID-19 [pandemic] revealed the severity of the brokenness.” [P1008]

### Critical SDOH in Detroit

Participants’ responses were most strongly linked to seven of the nine SDOH in the Conceptual Model of Community-Based Solutions (Fig. 1). They reported additional local SDOH like political representation, white flight from urban neighborhoods, the digital divide, and the need for more Black physicians and health care professionals. See Table 1 for additional detail and participant quotes.

**Table 1.** Critical SDOH in Detroit

SDOH domain	Key responses from summaries	Example quote
Education	Low literacy rates; public education via a biased lens on race and privilege; absence of a strong health curriculum. Also, concerns raised about individual-level health literacy, knowledge, and self-advocacy.	…there was a documentary from [local literacy coalition] I believe, explaining about how [local public school system] had such a low reading level when they got out of high school that it was actually impeding their ability to maintain a job, just be able to live right? So, if we aren’t setting up the poorest of our, you know, children up to be successful in just existing, right, we’re going to continue to see a widening of our lower classes… it goes back to the systems that are being put into place that make it really difficult for people to get ahead.” [P1014]
Employment	Unemployment/underemployment; the need for more effective workforce development strategies and elimination of barriers to career opportunities.	“Employment policies are huge because it’s through the economics that people are able to eventually buy better food, live somewhere they’re not living…Most people can’t afford to be in a workforce development program for 3 to 6 months because they don’t have the resources to pay their bills at the same time to do all of the work, let alone the internships - those programs cost. So, what does that mean? We set up workforce development programs that are inherently gonna fail or not hit their - hit their goals.” [P1016]
Housing	Housing instability and homelessness; lack of affordable housing; limited housing inventory for low-income families.	“If you don’t have housing security, it’s going to be a challenge. If you don’t know where you’re sleeping, and if you’re couch surfing, and people are always threatening to put you out, saying 'You can only stay here tonight. You gotta find somewhere tomorrow.' I can’t begin to focus on nothing else except where am I going to go? Right? So, the housing issue, and it’s not going away. We do not have quality affordable housing in the city of Detroit.” [P1006]
Income and wealth	The role of capitalism in uneven income distribution; the current economy and inflation; the need for increased sources of public monetary assistance.	“…if you just give poor people money, it helps a lot and, so, kind of just hone in on that one. I used to think like, oh, if you intervene early with children, maybe then you can kind of like, break the cycle of intergenerational everything. But just get poor people money. I really think that all the foundations that have all the money, that keep doling it out to nonprofits…I really think that they should just close their doors and give all their money to poor people, and then see what happens, because I think it will fix a lot of things.” [P1015]
Physical environment	Urban blight and the need to secure vacant, abandoned houses that negatively affect public safety, psychological well-being, and social cohesion; infrastructure issues related to water quality, air pollution originating from sources such as transportation emissions; environmental racism and the need for environmental justice efforts.	“…the financial crisis came. People started losing their houses… so those houses became vacant and empty and dangerous, open and dangerous….And so, we started boarding those up. We started securing them. We were lucky that we were able to work on it…I remember when I used to just ride through and survey our neighborhood it would take me a whole day to recover from seeing the blight, the disorganization. The abandonment! Psychologically, it did something to me, and I’m sure it did it to many, many people with regard to their sense of safety, belonging.” [P1001]
Public safety	Interpersonal and community level violence; the effects of violence exposure on mental health; the long-lasting effects of fear and chronic stress that may be generationally transferred; ineffectiveness of local police and public safety officers.	“That safety is important. That safety kills dreams when you don’t have it, and when I see violence in my home, and the pillars who are to protect me are broken and fragile and they live in fear, and then they transfer their fear to me. So now I don’t just bear my fear. I bear my mom’s fear. I bear my grandma’s fear.” [P1005]
Transportation	Need for improvements to the local transportation system, described as “dire”; long wait times for medical transportation services; limited public options; safety concerns as a limitation of using public transportation after dark.	“You know, we are in Detroit, and we do have a high crime rate that seems to be increasing …it is definitely something that I think is impacting people. And I say that to say that it makes people not want to leave their homes. So that means that if they have a later doctor’s appointment at 4 o’clock, and they have to take public transportation or something to that effect, they may not go to that appointment because they’ll be coming back home in the dark where it’s potentially unsafe.” [P1011]

### Social-Ecological Influences

Participants discussed all levels of influence in the SEM (Fig. 2) and offered their perspectives on those levels with greatest impact on health equity and outcomes.

#### Societal

Participants acknowledged the driving force and impact of overarching systems, such as racism, capitalism, and sexism on health. Some participants suggested a need for targeted messaging in the health care context regarding data and “lessons learned” about sexism, ageism, ableism, and other systems.

“… number one, we need to address the elephant in our country, and that elephant is institutional racism. Institutional racism has a direct impact … on health equity. . . . It exists in our systems.” [P1008]

#### Policy

Policy was the most frequently referenced level of influence on health. Participants noted policy change is important for impact and that individuals working toward health equity should be engaged and observant at some policy level. Participants emphasized the importance of voting rights, noted the need for community voice to create effective public policy and the urgency of creating change when the political climate allows for it. The role of policy was affirmed in establishing community standards to counter institutional racism. Participants further asserted the importance of ensuring that policy makers and legislators are accountable and responsive to communities rather than corporations.

“You might want to add voting rights [to policy], too, because to me that is, that is under warfare. And how do you change public policy if you are limited in your ability to vote?” [P1010]

“So public policy, I think, is key because if you can garner people, you can do advocacy and that draws attention and change. We don’t know the power that we have in public policy. We can put people up and we can bring them down. They have to listen to us. We have the power. If we vote for somebody, you’re gonna listen to me. You’re gonna listen to me. And that’s the way it should be. Public policy is really key.” [P1008]

#### Institutional

The potentially powerful influence of institutions on health equity included examples of the auto industry and health care system as two of the largest industries in Michigan with tremendous influence and economic impact. It was hypothesized that if such industries and corporations in general addressed SDOH, other entities would follow.

“I think that institutional level is probably the greatest influence here in the city of Detroit…You have these giants of industry… the health systems are also institutions. I think that those are the two biggest industries, if you will, in the state of Michigan, health care and the auto makers. A lot of things are influenced by what they’re doing, what’s going on with them. …If they were creative and rethought some things, we would probably see a lot of the issues that we were talking about on the other diagram with housing and wealth and employment, probably, be reduced. [P1017]

Institutional influence was also discussed in terms of “turf wars” between non-profit organizations, which can impede synergy and collaborations.

#### Interpersonal

Participants noted the importance of families, especially parents and grandparents, in shaping one’s beliefs, attitudes, and behavior. In the health care context, the need for physicians and health care providers to be less authoritative, demonstrate respect, improve communication, and increase shared decision making was discussed as part of interpersonal influence.

“I think [doctors] need to humble themselves. I think many of them are in the position of having an authoritative viewpoint, like, ‘I know what’s best for you and I’m going to dictate that to you.’ So, most of the time, you go to your doctor, they look at your symptoms or whatever, and then they say … authoritatively, do this and do that, no conversation unless you just learn to be advocating, push back, or whatever.” [P1001]

#### Individual

A number of individual-level factors were discussed as important, including one’s knowledge of history and cultural beliefs, avoidance of health care due to fear (especially in impoverished areas), health literacy, awareness of family history, health information seeking, and active involvement in one’s health care. One participant described this in terms of race-based medicine, or the use of race and potentially biased disease risk algorithms in clinical decision making.

“If everybody knew to go in and ask your doctor when he’s doing his different tests….’Hey, you practice race- based medicine? Did you make a correction on my number when I took my test? I want to know, Doc.’ ‘Oh, yeah, I did.’ ‘Well, don’t do that. That’s unacceptable. I don’t accept that…I need [care] based on this number, now, the real number. How would you have treated me? I ain’t gonna let you put the- put the adjustment on me and then tell me I’m fine.’ So, if we began to educate and everybody in the community knew how to be their own best advocate, the health system would kind of come to a screeching halt a little bit because people would be challenging all these different things that are being put upon them. [P1006]”

### Health Equity Strategies

Numerous health equity strategies were described, including several that have been implemented locally. Strategies were grouped into the following categories: 1) community engagement and advocacy, 2) housing and built environment, 3) neighborhood-based health care innovation, 4) whole person health, 5) food security, and 6) digital health (Table 2). Participants noted the importance of both identifying community-based health equity practices determined to be successful outside of Detroit that could be adapted within the city and improving dissemination of effective practices.

**Table 2.** Health Equity Strategies

Strategy	Summary of participant responses
Community engagement and advocacy	• Gender-specific strategies, specifically outreach to men by men. • Peer-to-peer outreach and education, particularly among youth. • Engagement with faith-based organizations (FBOs) and FBO collaboration with mental health services. • Structured engagement of older adults and youth (in community and school settings). • Promotion of volunteer opportunities with “on-the-ground” organizations. • Training of community members as champions/advocates and provision of knowledge and tools. • Collaboration with university student organizations, providing students with health outreach training and certification, especially Black students. • Regular convening of community members to discuss problems, deliberate, problem solve, and collaborate. • Multi-sector collaboration focused on policies, systems, and environments.
Housing and built environment	• Affordable housing with a focus on programs to build renter equity and energy sustainability. • Initiatives to board up and secure vacant/abandoned properties. • Support of community-based organizations (CBOs) and FBOs to build up vacant homes in occupied areas. • Home repair programs. • Property tax exemption support. • Leveraging of Detroit’s American Rescue Plan Act funding to support affordable housing developers. • Development of green infrastructure.
Neighborhood-based health care innovation	• Mobile health units. • Pop-up services (e.g., groceries, vaccines, mental health services) in communities where those services are not part of the built environment. • School health clinics as community health hubs. • Community health worker (CHW) networks to facilitate peer-to-peer support. • Community-based health care navigation. • CHW infrastructure to support the viability and sustainability of the role.
Whole person health	• Resource navigation and care management for community residents. • Resource mapping. • Wrap-around care that addresses gaps in social needs and improves quality of life. • FBO/CBO creation of transitional housing to provide wrap-around services that in 6 months position tenants to be able to support community needs.
Food security	• Seasonal farmer’s markets. • Community grocer review program to highlight and develop food sources in neighborhoods. • Programs focused on youth, health and nutrition education, and food-based entrepreneurship.
Digital health	• Creation of communication hubs via websites and social media to address physical activity and nutrition. • Increased accessibility of older adult-friendly devices to bridge the digital divide and connect older adults to social and digital resources. • Drop-in open computer labs.

### Short-Term Impact of RQA on Coalition Activities

As described above, data collection and analyses took about six months to complete. D-CHEA meets approximately monthly to engage in dialogue around health equity and program planning, as well as receive formal presentations related to the coalition’s mission (e.g., presentation of local data, dissemination of information on local equity initiatives) and updates of activities of member organizations. Following data analysis, study findings were shared with D-CHEA via PowerPoint presentation over the course of two meetings. These presentations stimulated robust discussion of the ways in which findings might inform the coalitions’ priorities. Further, a print version of the draft research report was shared with D-CHEA members to ensure that the summaries accurately reflected their views. Although informal, this approach was consistent with member checking, the process of soliciting feedback from one’s participants or stakeholders about study data and its interpretation and validating qualitative results and their trustworthiness.^22^ Moltulksy et al.^22^ note that providing participants with a summary of themes or interpretations, preliminary analyses of findings across cases, or even drafts of the entire research report and requesting response or feedback is a common approach to member checking. Other authors have concurred, stating, “The findings are what audience members will actually read, and therefore the weight of the findings is far greater than the transcripts. Once the researcher has a strong draft of the findings (including all direct quotations), participants should be asked to provide input. This approach allows participants’ expertise to be utilized in a way that benefits the final product. Participants are also much likelier to read a findings section as they can see the integration of their story or experience with other participants’ stories.”^23^ It should be reiterated here that, while our process was consistent with a widely accepted member checking approach, it was informal in practice, and we did not actively solicit feedback beyond the presentations described above.

## Discussion

This study explored the perspectives on health equity among members of the D-CHEA community coalition in Detroit. A key part of this work was the use of RQA.^18–20^ This time-sensitive approach is one that may be well-aligned with the processes of an academic-community coalition in which members hold different expectations for process and timeline.

A key finding was that most responses included some reference to equal access to opportunities to have a healthy life. Additionally, participants stressed the importance of individual choice in pursuing those opportunities as well as the ability to advocate on behalf of oneself and others. This emphasis suggests that when addressing health equity at the individual level, solutions cannot be prescriptive. Rather, they must be person-centered, so individual choice and preferences are respected. Participants shared their views on the most urgent health problems in Detroit and, in addition to local pressing health issues, participants described SDOH as health problems that need to be addressed especially poverty, low income, un- and underemployment, and the psychological and physiological pathways between SDOH exposures and health outcomes. These responses may reflect the growing awareness of SDOH and how social-ecological factors are integral to health, an awareness potentially heightened by the COVID-19 pandemic.^24^

When asked about the most urgent SDOH in the city, participants cited and provided examples related to housing, education, the physical and social environment, and income. Here, the relevance of local perspectives and context becomes apparent. For example, while the “physical environment” can refer to many elements of the built environment, several participant responses revealed that in Detroit, a major issue is the presence of blighted homes. Currently, 72.1% of Detroit residents report blight in their community, defined as a building or property visibly deteriorating in a way that suggests long-term neglect in their neighborhoods.^25^ Some of these forces were explained by participants, including white flight and suburbanization in the 1960s and 1970s that diminished the city’s tax base, overall population decline, the foreclosure crisis during which more than 100,000 Detroit homes were repossessed between 2012 and 2017, and Detroit’s bankruptcy in 2013.^26,27^ This is an example of how community-grounded perspectives and local context shed light on the nature of a problem, the forces that contribute to the problem, and opportunities for solutions.

When asked what social-ecological factors have the greatest influence on health equity and outcomes in Detroit, participants discussed all levels of the model and provided local context. For example, at the institutional level, corporate involvement in community health was mentioned, with specific reference to the automotive industry, a sector that has shaped not only the region’s financial and physical infrastructure but also its social and cultural norms—with a significant environmental impact. This is underscored by a recent study revealing the auto industry contributes a total of $225 billion to Michigan’s economy and supports 712,000 jobs.^28^ Such specific examples are useful to coalitions to inform their decision-making and goal-setting.

Participants further described strategies toward achieving health equity, based primarily on past or ongoing initiatives. Strategies were divided across five categories, with community engagement/advocacy, and housing/built environment as the leading two. Identifying these strategies is particularly important for D-CHEA because it highlights existing strengths that can be leveraged, thereby allowing the coalition to build on strengths and resources in the community, a core principle of community-based participatory research (CBPR).^29^

Study findings guided the work of the coalition in several ways. First, it was important to coalition members that the group have a shared definition of health equity since the coalition was convened to address this very topic. The group also thought it vital that all members have the opportunity to contribute to this definition so that it could reflect members’ diverse experiences. There was broad consensus and satisfaction across D-CHEA that the emergent definition of health equity was consistent with previous definitions but also nuanced, embodying Detroit’s social, historical, and political complexities. Second, the depth of participant responses related to social determinants of health informed D-CHEA’s first pilot project to standardize the collection of data on the social needs of patients in a clinical setting and develop a resource navigation model of patient referral to community-based service providers. In this project, social needs data were collected via the American Academy of Family Physicians (AAFP) Social Needs Screening Tool.^30^ Subsequent referrals are made by a certified community health worker already employed by one the D-CHEA partners. All D-CHEA members and organizations serve as consultants on this project and contribute to model development.

Current study findings also demonstrated the utility of the interview questions, which were included in another D-CHEA project: community listening sessions conducted in partnership with one D-CHEA member, a Detroit-based community development organization (CDO) serving three zip codes in the city. CDOs are place-based, professional, non-profit organizations accountable to local stakeholders, who CDOs convene for joint planning, problem solving and advocacy around a wide range of issues.^31^ CDOs also champion economic development in ways consistent with neighborhood priorities.^31^ Four listening sessions were conducted with 80 neighborhood residents, yielding rich, hyperlocal information about residents’ perceptions of the most urgent health inequities facing their community and the social drivers of those inequities, data that can be applied to the development of multilevel interventions.

This work is not without limitations. RQA is increasingly used, but there are concerns that a rapid approach might undermine scientific rigor and trustworthiness of data and findings.^32,33^ Furthermore, it is difficult to know how much time and effort RQA saved since it is impossible to know how long it would have taken to apply a more conventional approach to qualitative analysis and if the time saved was significant. However, at least one study has compared a traditional versus rapid approach to collecting, managing, and analyzing qualitative data and reported no difference between the two approaches in terms of rigor, including the credibility of findings, or the extent to which findings accurately portray respondents’ constructions.^34^ This same study revealed that the number of hours associated with the traditional approach (683 h) was 67% greater than the hours needed with the rapid approach (409.5 h).^34^ Finally, while our sample was purposeful and reflects the racial composition of Detroit, responses may not be fully representative of the broader population, particularly because most participants identified as female.

The study’s limitations are balanced by its strengths. While there are numerous health-focused community coalitions across the US, reports of best or evidence-based practices in establishing, advancing, and sustaining these groups are lacking. One practice highlighted in our work is the use of RQA. Other groups have used RQA to accelerate intervention work,^12^ but to our knowledge, this is the first study to apply RQA to facilitate cohesion and collaboration within a community coalition using well-established conceptual models of health equity that emphasize SDOH. RQA allowed D-CHEA to swiftly develop intellectually collaborative synergy by ensuring shared language and meanings, highlighting existing points of consensus, and bridging separate but related activities across coalition members. The adoption of a rapid approach also helped to convey a sense of shared urgency in addressing critical health inequities in Detroit, confirmed a shared mission, and likely increased member engagement, another dimension of collaborative synergy described in CCAT: the satisfaction, commitment, and participation of coalition members.^9^ The findings reported here reflect a wide range of topics and concerns that can be expanded upon using the academic literature, the gray literature (i.e., produced and published outside of traditional publishing channels), publicly available data archives, news media, and content experts, to name just a few sources. Even as more concise summaries, however, these findings are meaningful. They lay critical groundwork, rooted in participants’ lived experiences, for collaborative synergy through substantive relationship building and pooling of intellectual resources to ensure effective coalition assessment, planning, and implementation of strategies.^8^

### Health Equity Implications

The current study expands the concept of collaborative synergy necessary to the success of health equity coalitions. It further presents RQA as a promising approach to strengthening groups like D-CHEA that represent multi-sector coalitions, community-academic collaborations, and public/private partnerships. This time-efficient approach may be useful to similar urban coalitions and help them to collectively plan in an inclusive way that documents a wide range of perspectives and fosters stakeholder buy-in. It may be particularly useful in coalitions addressing the complexities of SDOH and provide them with a collaborative advantage in the development and implementation of multilevel interventions.

## Abbreviations Used

AAFP: American Academy of Family Physicians
ARPA: American Rescue Plan Act
CBO: Community-based organizations
CBPR: Community-based participatory research
CCAT: Community Coalition Action Theory
CDO: Community development organization
CHW: Community health worker
D-CHEA: Detroit Community Health Equity Alliance
FBO: Faith-based organization
LQBTQ: Lesbian, gay, bisexual, transgender and queer
MDHHS: Michigan Department of Health and Human Services
RQA: Rapid qualitative analysis
SDOH: Social determinants of health
SEM: Social Ecological Model
